# *N*-(Prop-2-yn-1-yl)-1,3-benzothia­zol-2-amine

**DOI:** 10.1107/S1600536811035136

**Published:** 2011-09-14

**Authors:** Alka Agarwal, Manavendra Kumar Singh, Suryabhan Singh, S. Bhattacharya, Satish K. Awasthi

**Affiliations:** a Department of Medicinal Chemistry, Institute of Medical Sciences, Banaras Hindu University, Varanasi 225 001, India; b Department of Chemistry, Banaras Hindu University, Varanasi 225 001, India; c Chemical Biology Laboratory, Department of Chemistry, University of Delhi 110 007, India

## Abstract

In the title compound, C_10_H_8_N_2_S, the 2-amino­benzothia­zole and propyne groups are not coplanar [dihedral angle = 71.51 (1)°]. The crystal structure is stabilized by strong inter­molecular N—H⋯N hydrogen bonds and C—H⋯C, C—H⋯π and F-type aromatic–aromatic [centroid–centroid distance = 3.7826 (12) Å] inter­actions are also observed.

## Related literature

For the biological activity of heterocyclic compounds, see: Xuan *et al.* (2001 ▶) and of benzothia­zole and benzimidazole compounds, see: Caroti *et al.* (1989 ▶); Paget *et al.* (1969 ▶); Da Settimo *et al.* (1992 ▶); Johnson *et al.* (2009 ▶); Kus *et al.* (1996 ▶). For N—H⋯N hydrogen bonding, see: Mingos & Braga (2004 ▶). For F-type aromatic–aromatic inter­actions, see: Zhang *et al.* (2010 ▶). For details of the synthesis, see: Lilienkampf *et al.* (2009 ▶). For recently reported small crystal structures and their anti­microbial activity, see: Singh, Agarwal & Awasthi (2011 ▶); Singh, Agarwal, Mahawar & Awasthi (2011 ▶); Awasthi *et al.* (2009 ▶).
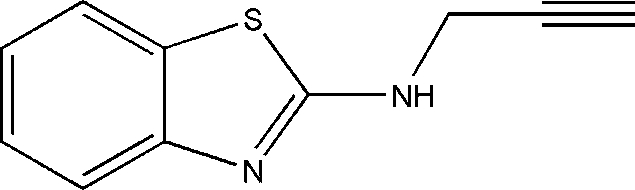


## Experimental

### 

#### Crystal data

C_10_H_8_N_2_S
*M* *_r_* = 188.25
Monoclinic, 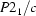

*a* = 6.8048 (4) Å
*b* = 8.6071 (5) Å
*c* = 15.8244 (8) Å
β = 99.445 (5)°
*V* = 914.26 (9) Å^3^
*Z* = 4
Mo *K*α radiation
μ = 0.30 mm^−1^
*T* = 293 K
0.40 × 0.39 × 0.38 mm

#### Data collection

Oxford Diffraction Xcalibur Eos diffractometer
Absorption correction: multi-scan (*CrysAlis PRO*; Oxford Diffraction, 2009 ▶) *T*_min_ = 0.771, *T*_max_ = 1.000
4188 measured reflections
2454 independent reflections
1428 reflections with *I* > 2σ(*I*)
*R*_int_ = 0.045

#### Refinement

*R*[*F* ^2^ > 2σ(*F* ^2^)] = 0.043
*wR*(*F* ^2^) = 0.131
*S* = 0.88
2454 reflections
128 parameters
H atoms treated by a mixture of independent and constrained refinement
Δρ_max_ = 0.17 e Å^−3^
Δρ_min_ = −0.27 e Å^−3^

### 

Data collection: *CrysAlis PRO* (Oxford Diffraction, 2009 ▶); cell refinement: *CrysAlis PRO*; data reduction: *CrysAlis PRO*; program(s) used to solve structure: *SHELXS97* (Sheldrick, 2008 ▶); program(s) used to refine structure: *SHELXL97* (Sheldrick, 2008 ▶); molecular graphics: *Mercury* (Macrae *et al.*, 2006 ▶); software used to prepare material for publication: *publCIF* (Westrip, 2010 ▶).

## Supplementary Material

Crystal structure: contains datablock(s) I, global. DOI: 10.1107/S1600536811035136/zj2020sup1.cif

Structure factors: contains datablock(s) I. DOI: 10.1107/S1600536811035136/zj2020Isup2.hkl

Supplementary material file. DOI: 10.1107/S1600536811035136/zj2020Isup3.cml

Additional supplementary materials:  crystallographic information; 3D view; checkCIF report

## Figures and Tables

**Table 1:** Hydrogen-bond geometry (Å, °) *Cg*1 is the centroid of the S1,C1,C6,N1,C7 ring.

*D*—H⋯*A*	*D*—H	H⋯*A*	*D*⋯*A*	*D*—H⋯*A*
N2—H*N*2⋯N1^i^	0.87 (3)	2.05 (3)	2.910 (2)	170 (2)
C8—H8*A*⋯C4^ii^	0.98	2.86	3.756 (3)	153 (1)
C10—H10⋯C1^iii^	0.89	2.87	3.687 (3)	153 (1)
C10—H10⋯C6^iii^	0.89	2.89	3.776 (3)	174 (1)
C10—H10⋯*Cg*1^iii^	0.89	2.74	3.548 (3)	151

Symmetry codes: (i) 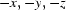; (ii) 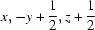; (iii) 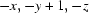.

**Fig. 1.**
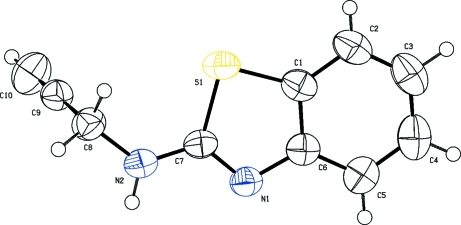
ORTEP diagram of the molecule with thermal ellipsoids drawn at 50% probability level Color code: White: C; yellow: S; blue: N; white: H.

**Fig. 2.**
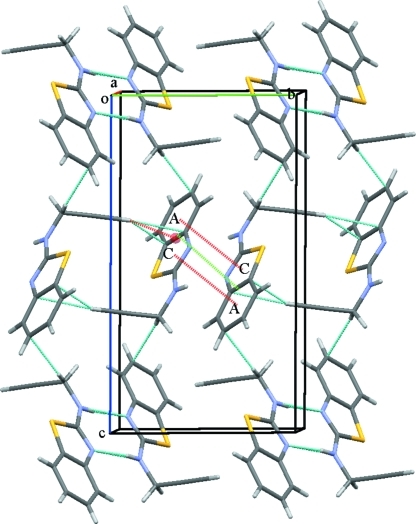
Packing diagram of molecule viewed through b plane showing supramolecular arrangement, Intermolecular N—H···N hydrogen bonding, C—H···π interactions and F-type aromatic-aromatic interaction.

**Fig. 3.**
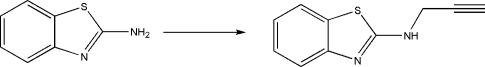
The formation of the title compound.

## supplementary crystallographic information

### Comment

Heterocyclic compounds containing nitrogen, sulfur, oxygen *etc* have immense importance especially in pharmaceutical industry. Most of the modern drugs contain one or more heteroatom in their scaffold. Further, oxidation of nitrogen in heterocycle plays key role in bioactivity of these scaffolds (Xuan, *et al.*, 2001). It is well documented that benzothiazole and benzimidazole derivatives show wide range of biological activities including antilipidemic (Caroti, *et al.*, 1989), antimicrobial (Kus, *et al.*, 1996), antiviral (Paget, *et al.*, 1969) anti-inflammatory and analgesic properties (Da Settimo, *et al.*, 1992). Moreover, 2-aminobenzimidazole/benzothiazole derivatives are common intermediate for the synthesis of various drugs. Anticancer properties of benzothiazole derivatives in cell based assays are also well documented (Johnson, *et al.*, 2009).Our research interest involves the antimicrobial activities of small molecule (Awasthi, *et al.*, 2009). Recently, we have reported several small crystal structures (Singh, Agarwal & Awasthi 2011, Singh, Agarwal, Mahawar *et al.*, 2011). We report here the crystal structure of *N*-(prop-2-yn-1-yl)-1,3-benzothiazol-2-amine (Figure 1).

In the title compound, the C7—N2 single bond (1.342 Å) is shorter than normal C—N bond (1.47 Å) suggesting a delocalized double bond in benzothiazole moiety. Further, N2—C8 bond (1.438 Å) is also shorter than a standard C—N bond distance due to delocalization of electrons. Again, it is evident from the crystal structure that the title compound is stabilized by strong intermolecular N—H···N hydrogen bonding as well as C—H···π interactions and aromatic π···π stacking interaction resulting in the formation of supramolecular arrangement in the cystal as seen in the crystal packing along *b* axis (Figure 2, Table 1). The intermolecular hydrogen bond distance between N2···N1 (2.91 Å) is shorter than N···N average bond range 3.15 Å, suggesting strong hydrogen bonding (Mingos & Braga, 2004).

In the cystal packing two benzothiazole skeletons are arranged in an antiparallel fashion by F-type aromatic–aromatic interactions and form a dimer, the ring A and C of an benzothiazole skeleton stacks with the ring C and A of another adjacent benzothiazole skeleton, respectively. The distance of CgA and CgC is 3.783 Å, where CgA and CgC are the center of ring A and C, respectively and the centroid - centroid distance between two adjacent benzothiazole ring is 3.879Å (Zhang *et al.*, 2010). 2-Aminobenzothiazole and propyne group are not co-planar with a dihedral angle of 71.51°. The torsion angles of C7—N2—C8—C9 and C10—C9—C8—N2 are found 91.1 (3) and 44 (7)° respectively. The CCDC No. of the crystal is 806158.

### Experimental

The synthesis of the title compound was carried out according to the published procedure (Lilienkampf, *et al.*, 2009). Briefly, to a solution of 2-aminobenzothaizole (0.90 g, 6 mmol) in dry acetone was added anhydrous K_2_CO_3_ (4.97 g m, 32 mmol) and reaction mixture was further refluxed for 15–30 minutes. Subsequently, KI (0.50 g m, 3 mmol) and propargyl bromide (0.64 ml, 7.2 mmol) were added and further refluxed the reaction mixture for 18 hrs. The reaction mixture was cooled, filtered, and the filtrate was evaporated *in vacuo* to give the product which was purified by column chromatography using hexane and dichloromethane (65:35) as eluent. The product was crystallized from hexane:dichloromethane (1:1). The light pink colored crystals were obtained by slow evaporation of solvent at room temperature in several days. Yield = 20%. MS (Macromass G) m/*z* = 188 (*M*^+^), *R*_f_ = 0.59 (98:2, CH_2_Cl_2_: MeOH), m.p.= 215°, Elemental analysis (Perkin –Elmer 240 C elemental analyzer) Calculated for: C_10_H_8_N_2_S (%) C– 63.8, H-4.2, N -14.9, S-17.1, found C-63.9, H-4.5, N -14.7, S-16.9. ^1^H NMR (CDCl_3_), 8.35 (s, 1H, NH), 7.71–7.68 (m, 1H), 7.44–7.42(m, 1H), 7.26–7.21 (m, 1H), 7.07–7.02(m, 1H), 4.18–4.17(m, 2H, CH_2_) 3.21 (s, 1H, CH).

### Refinement

All H atoms were located from difference Fourier map (range of C—H = 0.87 - 0.98 Å and N–H = 0.87 Å) and allowed to refine freely.

### Crystal data

**Table:**

C_10_H_8_N_2_S	*F*(000) = 392
*M**_r_* = 188.25	*D*_x_ = 1.368 Mg m^−^^3^
Monoclinic, *P*2_1_/*c*	Mo *K*α radiation, λ = 0.71073 Å
*a* = 6.8048 (4) Å	Cell parameters from 1697 reflections
*b* = 8.6071 (5) Å	θ = 3.5–29.1°
*c* = 15.8244 (8) Å	µ = 0.30 mm^−^^1^
β = 99.445 (5)°	*T* = 293 K
*V* = 914.26 (9) Å^3^	Block, light pink
*Z* = 4	0.40 × 0.39 × 0.38 mm

### Data collection

**Table:**

Oxford Diffraction Xcalibur Eos diffractometer	2454 independent reflections
Radiation source: fine-focus sealed tube	1428 reflections with *I* > 2σ(*I*)
graphite	*R*_int_ = 0.045
ω scans	θ_max_ = 29.1°, θ_min_ = 3.5°
Absorption correction: multi-scan (*CrysAlis PRO*; Oxford Diffraction, 2009)	*h* = −9→5
*T*_min_ = 0.771, *T*_max_ = 1.000	*k* = −10→9
4188 measured reflections	*l* = −19→19

### Refinement

**Table:**

Refinement on *F*^2^	Primary atom site location: structure-invariant direct methods
Least-squares matrix: full	Secondary atom site location: difference Fourier map
*R*[*F*^2^ > 2σ(*F*^2^)] = 0.043	Hydrogen site location: inferred from neighbouring sites
*wR*(*F*^2^) = 0.131	H atoms treated by a mixture of independent and constrained refinement
*S* = 0.88	*w* = 1/[σ^2^(*F*_o_^2^) + (0.0804*P*)^2^] where *P* = (*F*_o_^2^ + 2*F*_c_^2^)/3
2454 reflections	(Δ/σ)_max_ = 0.05
128 parameters	Δρ_max_ = 0.17 e Å^−^^3^
0 restraints	Δρ_min_ = −0.27 e Å^−^^3^

### Special details

**Table:**

Geometry. All s.u.'s (except the s.u. in the dihedral angle between two l.s. planes) are estimated using the full covariance matrix. The cell s.u.'s are taken into account individually in the estimation of s.u.'s in distances, angles and torsion angles; correlations between s.u.'s in cell parameters are only used when they are defined by crystal symmetry. An approximate (isotropic) treatment of cell s.u.'s is used for estimating s.u.'s involving l.s. planes.
Refinement. Refinement of *F*^2^ against ALL reflections. The weighted *R*-factor *wR* and goodness of fit *S* are based on *F*^2^, conventional *R*-factors *R* are based on *F*, with *F* set to zero for negative *F*^2^. The threshold expression of *F*^2^ > 2σ(*F*^2^) is used only for calculating *R*-factors(gt) *etc*. and is not relevant to the choice of reflections for refinement. *R*-factors based on *F*^2^ are statistically about twice as large as those based on *F*, and *R*- factors based on ALL data will be even larger.

### Fractional atomic coordinates and isotropic or equivalent isotropic displacement parameters (Å^2^)

**Table:**

	*x*	*y*	*z*	*U*_iso_*/*U*_eq_	
HN2	−0.004 (4)	0.093 (3)	0.0660 (17)	0.085 (8)*	
S1	0.42981 (8)	0.29415 (6)	0.04155 (3)	0.0554 (2)	
N1	0.2087 (2)	0.08399 (18)	−0.04719 (10)	0.0464 (4)	
N2	0.0971 (3)	0.1551 (2)	0.07821 (11)	0.0548 (5)	
C7	0.2251 (3)	0.1665 (2)	0.02239 (12)	0.0444 (5)	
C6	0.3657 (3)	0.1159 (2)	−0.09078 (12)	0.0457 (5)	
C9	−0.0017 (3)	0.3891 (3)	0.14573 (12)	0.0505 (5)	
C1	0.5014 (3)	0.2280 (2)	−0.05259 (13)	0.0497 (5)	
C8	0.1095 (3)	0.2429 (3)	0.15623 (14)	0.0541 (5)	
H8A	0.250 (2)	0.2658 (4)	0.1781 (3)	0.065*	
H8B	0.0582 (7)	0.1790 (9)	0.1993 (6)	0.065*	
C2	0.6662 (4)	0.2711 (3)	−0.08909 (17)	0.0638 (7)	
H2	0.750 (2)	0.3410 (19)	−0.0650 (7)	0.077*	
C5	0.3948 (3)	0.0473 (3)	−0.16713 (14)	0.0577 (6)	
H5	0.306 (2)	−0.0263 (18)	−0.1935 (7)	0.069*	
C3	0.6923 (4)	0.2005 (3)	−0.16390 (17)	0.0705 (7)	
H3	0.805 (3)	0.2274 (8)	−0.1895 (7)	0.085*	
C4	0.5588 (4)	0.0906 (3)	−0.20341 (16)	0.0674 (7)	
H4	0.5794 (6)	0.0458 (12)	−0.2544 (14)	0.081*	
C10	−0.0941 (4)	0.5029 (3)	0.13609 (15)	0.0737 (7)	
H10	−0.165 (2)	0.591 (2)	0.1287 (3)	0.088*	

### Atomic displacement parameters (Å^2^)

**Table:**

	*U*^11^	*U*^22^	*U*^33^	*U*^12^	*U*^13^	*U*^23^
S1	0.0584 (4)	0.0468 (4)	0.0591 (4)	−0.0137 (2)	0.0038 (3)	−0.0032 (2)
N1	0.0444 (9)	0.0428 (9)	0.0508 (9)	−0.0020 (7)	0.0046 (7)	−0.0031 (7)
N2	0.0561 (12)	0.0534 (11)	0.0553 (10)	−0.0136 (9)	0.0101 (9)	−0.0129 (9)
C7	0.0454 (11)	0.0370 (10)	0.0484 (10)	−0.0012 (9)	0.0009 (8)	0.0009 (8)
C6	0.0435 (11)	0.0409 (11)	0.0506 (11)	0.0074 (9)	0.0010 (9)	0.0062 (9)
C9	0.0494 (12)	0.0545 (13)	0.0476 (11)	−0.0088 (10)	0.0081 (9)	−0.0043 (9)
C1	0.0482 (11)	0.0429 (12)	0.0567 (12)	0.0018 (9)	0.0045 (9)	0.0114 (9)
C8	0.0607 (13)	0.0532 (13)	0.0480 (11)	−0.0043 (11)	0.0078 (10)	−0.0035 (10)
C2	0.0572 (14)	0.0577 (14)	0.0773 (17)	−0.0066 (11)	0.0130 (12)	0.0125 (12)
C5	0.0582 (13)	0.0584 (14)	0.0544 (12)	0.0092 (11)	0.0030 (10)	0.0013 (10)
C3	0.0617 (15)	0.0776 (19)	0.0761 (17)	0.0052 (13)	0.0227 (13)	0.0242 (14)
C4	0.0686 (16)	0.0786 (17)	0.0572 (13)	0.0226 (14)	0.0164 (12)	0.0117 (12)
C10	0.0827 (17)	0.0700 (17)	0.0664 (15)	0.0148 (15)	0.0062 (13)	−0.0025 (13)

### Geometric parameters (Å, °)

**Table:**

S1—C1	1.738 (2)	C1—C2	1.394 (3)
S1—C7	1.7612 (19)	C8—H8A	0.9843
N1—C7	1.299 (2)	C8—H8B	0.9843
N1—C6	1.391 (2)	C2—C3	1.368 (3)
N2—C7	1.342 (3)	C2—H2	0.8728
N2—C8	1.438 (3)	C5—C4	1.388 (3)
N2—HN2	0.87 (3)	C5—H5	0.9257
C6—C5	1.388 (3)	C3—C4	1.387 (3)
C6—C1	1.402 (3)	C3—H3	0.9510
C9—C10	1.160 (3)	C4—H4	0.9255
C9—C8	1.464 (3)	C10—H10	0.8942
			
C1—S1—C7	88.46 (10)	C9—C8—H8A	108.9
C7—N1—C6	110.27 (17)	N2—C8—H8B	108.9
C7—N2—C8	125.02 (19)	C9—C8—H8B	108.9
C7—N2—HN2	118.2 (16)	H8A—C8—H8B	107.7
C8—N2—HN2	116.7 (16)	C3—C2—C1	117.9 (2)
N1—C7—N2	122.92 (18)	C3—C2—H2	121.1
N1—C7—S1	116.23 (15)	C1—C2—H2	121.1
N2—C7—S1	120.83 (15)	C6—C5—C4	119.0 (2)
C5—C6—N1	125.34 (19)	C6—C5—H5	120.5
C5—C6—C1	119.37 (19)	C4—C5—H5	120.5
N1—C6—C1	115.29 (17)	C2—C3—C4	121.7 (2)
C10—C9—C8	178.2 (2)	C2—C3—H3	119.2
C2—C1—C6	121.5 (2)	C4—C3—H3	119.2
C2—C1—S1	128.74 (19)	C3—C4—C5	120.6 (2)
C6—C1—S1	109.74 (14)	C3—C4—H4	119.7
N2—C8—C9	113.45 (18)	C5—C4—H4	119.7
N2—C8—H8A	108.9	C9—C10—H10	180.0
			
C6—N1—C7—N2	−177.76 (18)	C7—S1—C1—C2	−179.2 (2)
C6—N1—C7—S1	1.1 (2)	C7—S1—C1—C6	0.15 (15)
C8—N2—C7—N1	179.58 (19)	C7—N2—C8—C9	91.1 (3)
C8—N2—C7—S1	0.7 (3)	C10—C9—C8—N2	44 (7)
C1—S1—C7—N1	−0.77 (16)	C6—C1—C2—C3	0.0 (3)
C1—S1—C7—N2	178.15 (17)	S1—C1—C2—C3	179.24 (17)
C7—N1—C6—C5	179.50 (18)	N1—C6—C5—C4	−179.97 (17)
C7—N1—C6—C1	−1.0 (2)	C1—C6—C5—C4	0.6 (3)
C5—C6—C1—C2	−0.7 (3)	C1—C2—C3—C4	0.8 (4)
N1—C6—C1—C2	179.82 (18)	C2—C3—C4—C5	−0.9 (3)
C5—C6—C1—S1	179.97 (15)	C6—C5—C4—C3	0.2 (3)
N1—C6—C1—S1	0.4 (2)		

### Hydrogen-bond geometry (Å, °)

**Table:**

Cg1 is the centroid of the S1,C1,C6,N1,C7 ring.

**Table:**

*D*—H···*A*	*D*—H	H···*A*	*D*···*A*	*D*—H···*A*
N2—HN2···N1^i^	0.87 (3)	2.05 (3)	2.910 (2)	170 (2)
C8—H8A···C4^ii^	0.98	2.86	3.756 (3)	153.(1)
C10—H10···C1^iii^	0.89	2.87	3.687 (3)	153 (1)
C10—H10···C6^iii^	0.89	2.89	3.776 (3)	174 (1)
C10—H10···Cg1^iii^	0.89	2.74	3.548 (3)	151

Symmetry codes: (i) −*x*, −*y*, −*z*; (ii) *x*, −*y*+1/2, *z*+1/2; (iii) −*x*, −*y*+1, −*z*.
